# Evidence-Based Percutaneous Closure of the Left Atrial Appendage in Patients with Atrial Fibrillation

**DOI:** 10.2174/157340312801215827

**Published:** 2012-02

**Authors:** Sílvio Leal, Raúl Moreno, Manuel de Sousa Almeida, José Aniceto Silva, José L López-Sendón

**Affiliations:** 1 Cardiovascular Intervention Unit, Cardiology Service, Centro Hospitalar de Lisboa Ocidental, Lisbon, Portugal; 2 Interventional Cardiology Unit, Cardiology Service, Hospital Universitario La Paz, Madrid, Spain

**Keywords:** Atrial fibrillation, left atrial appendage, oral anticoagulation, percutaneous closure devices, stroke.

## Abstract

Atrial fibrillation is the most common cardiac arrhythmia, and its prevalence is increasing. Cardioembolic stroke, most of the times secondary to thrombus formation in the left atrial appendage, is its most feared and life threatening consequence. Oral anticoagulation with vitamin-K-antagonists is currently the most used prophylaxis for stroke in patients with atrial fibrillation; unfortunately, its benefits are limited by a narrow therapeutic window and an increased risk for bleeding, making it often undesired. Percutaneous occlusion of the left atrial appendage is a novel alternative strategy for cardioembolic stroke prophylaxis in patients with atrial fibrillation at a high risk of stroke but with contraindication for long-term oral anticoagulation therapy. At present, several devices have been developed specifically for percutaneous occlusion of the left atrial appendage. Current results show good feasibility and efficacy for these devices, with a high rate of successful implantation, although also associated with the inherent potential periprocedural complications. This work reviews the current state of the art of percutaneous left atrial appendage closure for stroke prophylaxis in patients with atrial fibrillation.

## ATRIAL FIBRILLATION AND THROMBOGENESIS

Atrial fibrillation (AF) is the most common sustained cardiac arrhythmia and a major cause of morbidity and mortality. It presents a lifetime risk for development of 1 in 4 for persons over 40 years old, increasing its prevalence with age (up to 15% in octogenarians) just as its predisposing conditions like hypertension, diabetes, heart failure, and coronary heart disease [1, 2]. Cardioembolic stroke is the most serious and life threatening potential complication of AF, with an associated mortality up to 30% at 12 months and a 1 in 3 recurrence rate at 5 years [3, 4]. Atrial fibrillation is responsible for 25% of all ischemic strokes, occurring in 5% of non-anticoagulated patients every year. Stroke prophylaxis is therefore a socio-economically highly relevant component of management of AF [5, 6].

Left atrial appendage (LAA), a remnant of the embryonic left atrium, was demonstrated in echocardiographic and autopsy studies to be the source of thrombi in more than 90% of patients with nonvalvular AF [7-9]. This trabeculated blind spot presents a complex and highly variable anatomy, with a long, tubular, often multilobed body extending over the atrioventricular groove and left ventricular surface, and an oval-shaped ostium located between the left ventricle and the left upper pulmonary vein [10-12]. Thrombus formation in LAA presents a complex and not fully elucidated pathogenesis, expressed by Virchow’s triad of thrombogenesis [13, 14]: (1) abnormal changes of the vessel wall, translated by structural and functional changes of endothelial and endocardial cells probably related with a nonspecific inflammatory reaction; (2) abnormal blood flow, with stasis resultant from the diminished contractility of LAA combined with volume increase of LA and LAA – so called atrial remodeling; and (3) abnormal blood constituents, represented by activation of coagulation factors and platelets, most likely due to underlying cardiovascular disease [15-21].

## BENEFITS AND LIMITATIONS OF ORAL ANTICOAGULATION

Dose adjusted (INR 2.0-3.0) oral anticoagulation with vitamin-K-antagonists (VKA), warfarin being the most widely investigated drug, is currently the most established prophylaxis for stroke in AF. Despite its proven benefit - a reduction of cardioembolic events above 60% in patients with nonvalvular AF - oral anticoagulation with VKA remains underused in clinical practice. Only about 50% of warfarin-eligible patients actually are been treated with the drug, and those who are on this therapy are only about one-half of the time within the therapeutic range [22-24]. Several are the barriers to achieve a correct oral anticoagulation: (1) warfarin requires frequent laboratory monitoring; (2) it is often not well tolerated by patients; (3) its effectiveness varies due to interactions with foods, other medications and lifestyle; and (4) it has a very narrow therapeutic range, with high risk for bleeding complications that are potentially fatal. Bleeding risk is of great concern in the selection of patients to oral anticoagulation, especially in older patients and in those with higher CHADS2 score. It is important to note that patients at highest risk of stroke and, therefore, with greatest need for antithrombotic therapy are precisely those who experience more bleeding [25, 26]. 

Several pharmacological alternatives to VKA have been investigated. Antiplatelet therapy to prevent vascular events in AF patients presents disappointing results. Aspirin demonstrated a 22% relative risk reduction of stroke vs. placebo; however, when compared with warfarin showed a stroke rate 36% higher [27, 28]. These results have been recently confirmed in patients over 75, with aspirin being associated with a 52% higher rate of stroke and a similar incidence of major hemorrhage (2.0% versus 1.9% per year) compared to warfarin [29]. The combination of clopidogrel and aspirin demonstrated better results than aspirin alone but worse than warfarin in high risk AF patients, with a 44% higher rate of vascular events and a 30% increase in major bleeding [30, 31]. New anticoagulants have also been compared to VKA. Idraparinux was more effective than warfarin but was associated with a substantially higher risk of bleeding [32]. Ximelagatran appeared to be similar to warfarin with respect to efficacy and safety, but was found to be hepatotoxic [33]. In the RE-LY trial, two different doses of dabigatran, a direct oral thrombin inhibitor, were tested. The 110-mg dose was associated with similar rates of stroke and systemic embolism (9% reduction) and a 20% lower rate of major hemorrhage, while the 150-mg dose was associated with a 34% lower rate of stroke and systemic embolism but with a similar rate of major hemorrhage [34]. Rivaroxaban, an oral factor Xa inhibitor, demonstrated recently in the ROCKET-AF trial noninferiority to warfarin in stroke and systemic embolism (21% reduction) without significant difference in the risk of major bleeding [35]. Finally, apixaban, also a direct factor Xa inhibitor, showed in the AVERROES trial favorable results compared to aspirin in patients non suitable for warfarin (55% reduction in stroke and systemic embolism and 21% reduction in death, with similar major hemorrhage) [36], and in the ARISTOTLE trial superiority to warfarin in terms of stroke and systemic embolism (21% reduction), major hemorrhage (31% reduction) and death (11% reduction) [37]. Remarkably, the incidence of stroke and other embolic events in thousands of patients included in contemporary clinical trials is very low, less than 1.5% per year, in clear contrast with the incidence of embolic events in epidemiologic studies where patients with comorbidities and other possible factors for high risk are not excluded.

## LEFT ATRIAL APPENDAGE CLOSURE FOR STROKE RISK REDUCTION

**Fig. (1):**
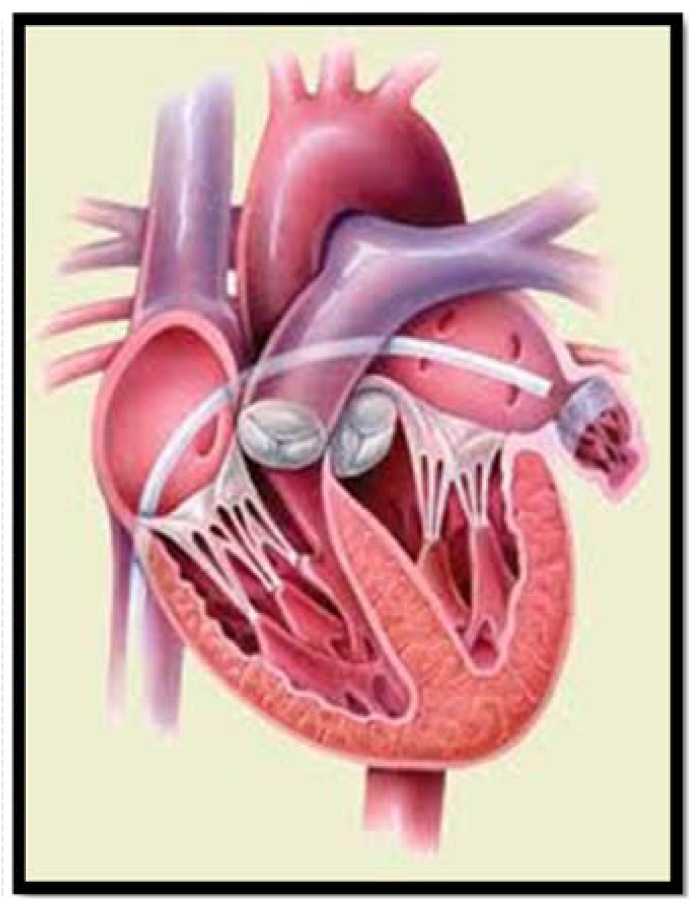
Percutaneous transseptal approach to left atrial appendage closure.

Patients with AF and high risk of stroke and with contraindications to long term OAC because of hemorrhage or other secondary effects may be candidates for alternative approaches that combine high efficacy in stroke prevention and low hemorrhagic risk. The central role of LAA as a source of embolism in patients with AF led to the hypothesis that resection or obliteration of the LAA, using either surgical or percutaneous techniques, might reduce the risk of stroke [38]. Surgical experience in the exclusion of LAA has more than 60 years of history, with first resection in human patients described in 1949 [39]. Its feasibility, safety and efficacy in stroke prevention in AF patients have been described in several retrospective analysis [40, 41], and the first published results in 2005 of the randomized LAA Occlusion Study (LAAOS) in patients undergoing elective coronary artery bypass graft surgery were also encouraging [42]. Nonetheless, the invasive nature of surgical or thoracoscopic LAA closure limited its general acceptance, other than as an adjunctive procedure in patients undergoing mitral valve surgery [43]. The development of less invasive percutaneous approaches to close the LAA by implantation of a mechanical device via a transseptal approach represents a clear step further in this field (Fig. **1**). 

### 1 The PLAATO System

The first device to be successfully developed for human use was the Percutaneous Atrial Appendage Transcatheter Occlusion (PLAATO) device (EV3, Inc., Plymouth, MN, USA), started in 2001 [44]. The PLAATO system consists of an implant device, a 15 to 32 mm diameter self-expanding nitinol cage, covered with an expanded polytetrafluoroethylene membrane that achieves complete closure of the LAA ostium, and a delivery catheter advanced to the LAA through a 12 F curved transseptal sheath. Anchoring of device is assisted by small hooklets along the struts and passing through the membrane. Several reports demonstrated efficacy in stroke prevention using PLAATO. The Percutaneous left atrial appendage transcatheter occlusion to prevent stroke in high-risk patients with non-rheumatic atrial fibrillation (PLAATO) study showed that, in 111 permanent or paroxysmal AF patients with contraindications for anticoagulation therapy and at least one additional risk factor for stroke, the PLAATO system was feasible and could be performed at acceptable risk [45]. In an average follow-up of 9.8 months, 1 patient needed cardiovascular surgery and suffered in-hospital neurological death, 3 patients underwent in-hospital pericardiocentesis due to a hemopericardium and 2 additional patients experienced stroke. The results at 5 years of this study reported 7 deaths, 5 major strokes, 3 minor strokes, 1 cardiac tamponade requiring surgery, 1 probable cerebral hemorrhage/death and 1 myocardial infarction in 64 patients [46]. The annualized stroke/transient ischemic attack rate was 3.8%, versus an anticipated rate by the CHADS2 scoring method of 6.6%/year [47]. Nevertheless, financial problems of the manufacturer and a significant rate of serious adverse events in the real world setting, including vessel perforation during vascular access, cardiac tamponade after transseptal puncture and device embolization, lead to discontinuation of the device in 2006 [48]. 

### 2 The WATCHMAN System

**Fig. (2):**
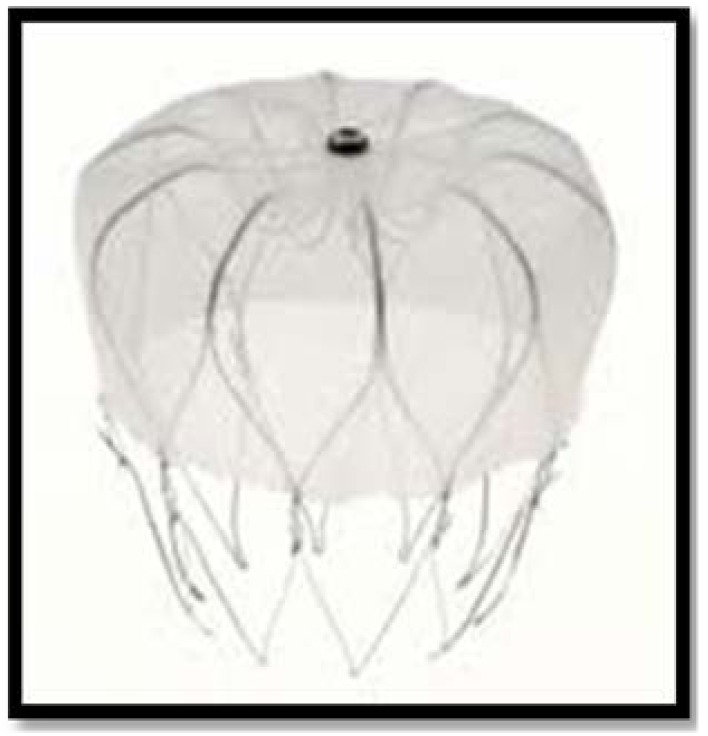
The WATCHMAN system.

The second device designed for percutaneous closure of LAA was the WATCHMAN Left Atrial Appendage System (Atritech, Inc., Plymouth, MN, USA), implanted since 2002 in Europe [49]. It is a three-part system consisting of a 12 F transseptal sheath, a delivery catheter and an implantable device, a self-expanding nitinol frame structure with fixation barbs and a permeable polyester cover available in diameters ranging from 21-33 mm (Fig. **2**). The Percutaneous closure of the left atrial appendage versus warfarin therapy for prevention of stroke in patients with atrial fibrillation (PROTECT-AF) trial [50, 51] was a multicentre, randomized non-inferiority trial designed to demonstrate safety and efficacy of the WATCHMAN device against warfarin therapy in nonvalvular AF patients with CHADS2 score >1. The study enrolled 707 long-term OAC eligible patients, randomly assigned in a 2:1 ratio to percutaneous closure of the LAA and subsequent discontinuation of warfarin or to dose adjusted warfarin treatment (INR 2.0-3.0). The technical success rate of implantation was of 91%. At 45 days, 86% of the 408 patients with an implanted device discontinued warfarin and initiated double antiplatelet therapy with aspirin and clopidogrel until completion of the 6-month follow-up visit, maintaining aspirin alone indefinitely from that date. At a mean follow-up of 18 months, patients who implanted the device showed a primary efficacy event rate (composite of stroke, cardiovascular death and systemic embolism) of 3.0 vs. 4.9% in control group (rate ratio 0.62, probability of noninferiority > 99.9%). Hemorrhagic stroke was less frequent in the intervention group (0.1 vs. 1.6%), as well as cardiovascular and all-cause mortality (0.7 vs. 2.7% and 3.0 vs. 

4.8%, respectively). Nevertheless, the rate of ischemic stroke was higher in the intervention group (2.2 vs. 1.6%), due to the occurrence of 5 periprocedural events, mainly air embolism. Safety endpoint results were also not so encouraging: 10.6% of the patients experienced serious procedural complications, with a 4.8% rate of major pericardial effusion. Device embolization occurred in 3 patients, with need for surgical removal in 2 of them; globally, 2.2% of attempted implantations resulted in cardiovascular surgical intervention due to device-related complications. No deaths were deemed related with the LAA closure device. 

### 3 The AMPLATZER CARDIAC PLUG System

**Fig. (3):**
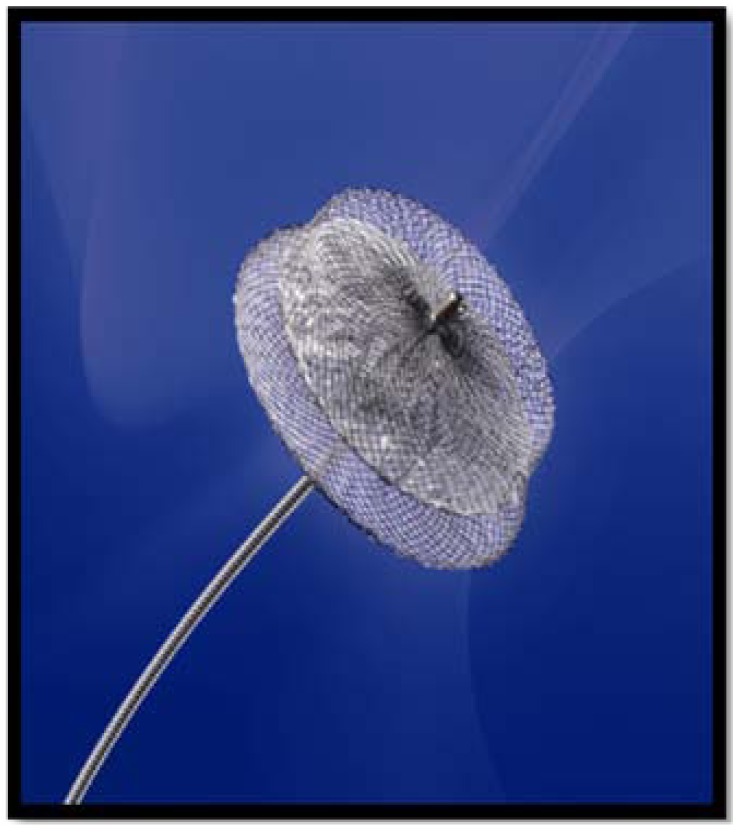
The AMPLATZER CARDIAC PLUG system.

**Fig. (4):**
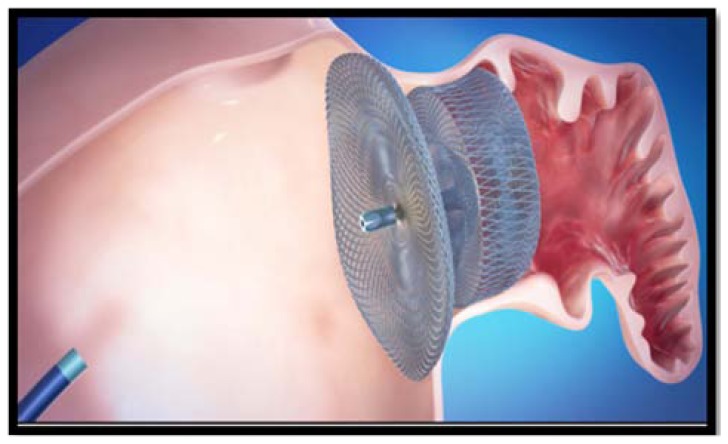
Schematic representation of the ideal positioning of AMPLATZER CARDIAC PLUG device in left atrial appendage.

The most recently developed percutaneous LAA closure device is the AMPLATZER Cardiac Plug (ACP) System (AGA, Inc., Minneapolis, MN, USA). The ACP is a 3-part system, consisting of a transseptal access sheath, a delivery catheter, and an implantable, self-expanding device (Fig. **3**). It evolved from the AMPLATZER double-disk septal occluders, devices designed for closure of atrial septal defects and patent foramen ovale but also used off label for LAA exclusion since 2002, practice discouraged by the results of a feasibility trial showing the occurrence of 1 embolization in 16 patients [52, 53]. The ACP implant, constructed from a nitinol mesh and a polyester patch, consists of a lobe and a disc connected by a central waist. The lobe has stabilizing hooks to improve device placement and fixation. The disc seals the outer shape of the LAA orifice in what has been termed the “pacifier principle” (Fig. **4**). The device diameter ranges from 16-30 mm referring to the lobe, being available in 8 sizes stepwise by 2 mm; the appropriate size is chosen to be 10% to 20% larger than the narrowest diameter of the LAA body 1 – 2 cm distal to the ostium, in way to have sufficient fixation of the lobe in the surrounding LAA myocardium for stable positioning of the device. The implant has threaded screw attachments for connection to the delivery and loading cable, as well as radio-opaque markers at each end and close to the stabilizing wires to support fluoroscopic positioning and, if necessary, retrieval and redeployment. The initial European experience with the ACP in AF patients was recently published [54]. In 137 out of 143 patients treated in 10 centers, LAA occlusion was attempted with a technical success rate of 96%. Serious complications were registered in 10 (7%) of the patients: 3 ischemic strokes, 2 device embolizations, both percutaneously recaptured, and 5 serious pericardial effusions. Minor complications were insignificant pericardial effusions in 4 patients, transient myocardial ischemia in 2 and loss of the implant in the venous system in 1 patient. In all patients dual platelet inhibition with aspirin and clopidogrel was recommended for 1-3 months, followed by aspirin alone for at least 5 months. A large-scale industry sponsored prospective registry study, started in December 2009, is ongoing and will expectedly clarify these preliminary results.

## CONTROVERSIES ABOUT PERCUTANEOUS LEFT ATRIAL APPENDAGE CLOSURE

Many doubts still persist regarding the risks and benefits of LAA closure for prevention of cardioembolic stroke in AF patients. Good feasibility was demonstrated for the three devices developed; however, the occurrence of major adverse events such as pericardial effusions with tamponade, periprocedural stroke, and device embolization was not rare and, although a direct comparison between devices is impossible with the available data, probably do not substantially differ between them. The key must pass through a criterious patient selection. First, only patients with contraindications or intolerance for anticoagulation and high risk of stroke may expectedly benefit from LAA occlusion – given that patients with a CHADS2 score of 1 have an annual estimated risk of stroke of 2.8%, similar to that registered with device implantation [47]. The ideal candidate profile may be the patient with high CHADS2 score, with stroke recurrence under OAC or with contraindication for OAC and without significant vascular disease. Second, an accurate image study prior to device implantation is essential [55, 56] – since that vulnerability of the LAA and the wash-out of pre-existing thrombi are two main sources of severe complications of the LAA occlusion procedure like pericardial effusion and stroke. Also the selection of the appropriate size of the device, one of the main difficulties of the implantation procedure, is a key to diminish complications; the high variability in terms of shape, volume (0.7-19.2 mL), length (16-51 mm), and size of the LAA orifice (5-40 mm) turn transesophageal or intracardiac echocardiographic guidance essential [57-59]. Finally, it can be expected that adverse event rates will decline with increased operator experience, being a comprehensive operator training prior to the first LAA device implantation strongly recommended. New studies with longer follow-up periods may also show more favorable results to LAA closure devices, since the high initial risk associated with implantation is offset by the progressive cumulative risk of chronic anticoagulation therapy. Periprocedural or long term anticoagulation after implantation in way to reduce ischemic risk must also be a matter of debate [60, 61]. Other concerns of percutaneous closure of LAA are related with the pathophysiological consequences of implanting a foreign body into the LAA, which remain to be fully elucidated. A potential late complication is fluid retention, since the LAA produces 30% of total cardiac atrial natriuretic factor (ANF) [62]. This finding, although described in surgical animal and human studies after bilateral appendagectomy, was however not shown in cases of isolated LAA exclusion [63-64]. Small iatrogenic atrial septal defects can also be created after transseptal puncture, although the most part disappear within 6 months. The risk of infection must be also of concern [65]. 

## CONCLUSION

Atrial fibrillation is a major cause of morbidity and mortality due to cardioembolic stroke. Due to its proven efficacy, chronic OAC is currently the prophylactic measure of choice in the most part of patients with AF, however not without a sometimes high bleeding risk. Since the LAA is the source of more than 90% of thrombi in nonvalvular AF patients, its occlusion may provide an alternative to chronic therapy with warfarin or new oral anticoagulants for stroke prophylaxis in accurately selected patients with simultaneous high embolic and hemorrhagic risk. The available percutaneous LAA closure devices share many similarities, showing good technical feasibility and an efficacy in stroke and cardiovascular death prevention non-inferior to OAC (not yet demonstrated in ACP device), but at cost of a 5-10% rate of serious periprocedural events - although without long-term sequelae for most patients. This limitation, although expected to decline with increasing implantation experience, need to be considered and overcome. Well designed clinical trials will be needed to establish the role of this new technique.
